# Anti-biofilm Activities from *Bergenia crassifolia* Leaves against *Streptococcus mutans*

**DOI:** 10.3389/fmicb.2017.01738

**Published:** 2017-09-13

**Authors:** Yucui Liu, Yanjie Xu, Qiuhang Song, Fei Wang, Luguo Sun, Lei Liu, Xiaoguang Yang, Jingwen Yi, Yongli Bao, Haifeng Ma, Honglan Huang, Chunlei Yu, Yanxin Huang, Yin Wu, Yuxin Li

**Affiliations:** ^1^National Engineering Laboratory for Druggable Gene and Protein Screening, Northeast Normal University Changchun, China; ^2^School of Life Sciences, Northeast Normal University Changchun, China; ^3^School of Physics, Northeast Normal University Changchun, China; ^4^People’s Liberation Army of China No.401 Hospital Qingdao, China; ^5^Department of Pathogenobiology, College of Basic Medical Sciences, Jilin University Changchun, China

**Keywords:** *Bergenia crassifolia*, biofilm formation, glucosyltransferase activity, extracellular polysaccharides, cytotoxicity

## Abstract

*Streptococcus mutans* has been reported as a primary cariogenic pathogen associated with dental caries. The bacteria can produce glucosyltransferases (Gtfs) to synthesize extracellular polysaccharides (EPSs) that are known as virulence factors for adherence and formation of biofilms. Therefore, an ideal inhibitor for dental caries is one that can inhibit planktonic bacteria growth and prevent biofilm formation. *Bergenia crassifolia* (L.), widely used as a folk medicine and tea beverage, has been reported to have a variety of bioactivities. The present study aimed to explore the effect of *B. crassifolia* (L.) leaf extracts on the biofilm of *Streptococcus mutans*. The *B. crassifolia* (L.) leaf extracts showed inhibitory effects by decreasing viability of bacteria within the biofilm, as evidenced by the XTT assay, live/dead staining assay and LDH activity assay, and could decrease the adherence property of *S. mutans* through inhibiting Gtfs to synthesize EPSs. In addition, the reduced quantity of EPSs and the inhibition of Gtfs were positively correlated with concentrations of test samples. Finally, the MTT assay showed that the extracts had no cytotoxicity against normal oral cells. In conclusion, the extracts and sub-extracts of *B. crassifolia* leaves were found to be antimicrobial and could reduce EPS synthesis by inhibiting activities of Gtfs to prevent bacterial adhesion and biofilm formation. Therefore, *B. crassifolia* leaves have potential to be developed as a drug to prevent and cure dental caries.

## Introduction

Dental caries is one of the most prevalent and costly infectious diseases in the world (Huang et al., 2016). Over 90% of the world’s population has been reported to suffer from it at least once over the course of a lifetime (Marchisio et al., 2010). Cariogenic biofilms play an important role in the development of caries. They develop as pathogens accumulated on tooth surfaces, forming highly structured microbial communities that are tightly adherent and enmeshed in an extracellular matrix (Davies, 2003; Paes Leme et al., 2006; Chen et al., 2016).

Within the complex oral microbiome, *Streptococcus mutans* is recognized as one of the primary pathogens of dental caries (Kaur et al., 2015). Furthermore, it has been demonstrated that the bacterium could cause endocarditis and intensify non-alcoholic steatohepatitis and ulcerative colitis (Nakano et al., 2009; Kojima et al., 2012; Naka et al., 2016). Although *S. mutans* is not always the most abundant organism among oral bacteria, it can rapidly orchestrate the formation of cariogenic biofilms (Bowen and Koo, 2011). *S. mutans* can produce at least three types of glucosyltransferases (Gtfs), GtfB, GtfC and GtfD, which can utilize dietary sucrose to synthesize extracellular polysaccharides (EPSs) (Bowen, 2002). GtfB catalyzes the synthesis of water-insoluble 1,3-linked glucan, and GtfC forms a water-soluble 1,6-linked glucan and a water-insoluble 1,3-linked glucan polymer; meanwhile, GtfD synthesizes a water-soluble 1,6-linked glucan (Hanada and Kuramitsu, 1988, 1989; Kopec et al., 1997). The water-insoluble glucan binds the bacterium for attachment to the tooth surface, while the water-soluble glucan may supply a source of metabolizable carbohydrate for plaque bacteria if nutrients become limited (Jenkinson and Lamont, 1997; Cross et al., 2007).

The World Health Organization (WHO) has reported that dental caries can be prevented in daily life. Traditional methods to prevent and control dental plaques consist of mechanical removal and the usage of broad-spectrum antibiotics (Chen et al., 2016). However, mechanical removal methods, such as brushing and flossing, are not effective for many individuals who lack the knowledge, skills or motivation, which limit their usage (Cummins, 2013). Chlorhexidine, one of the most broad spectrum antibiotics, is commonly used to prevent or cure infectious diseases and also to protect oral health by being used as a mouthwash. However, it may influence the oral physiological environment and even cause dysgeusia (Liu et al., 2011). As frequent use of chlorhexidine has been confirmed to increase the risk of drug resistance of bacteria not only in the mouth but also in other parts of body and cause cell damage (El-Moug et al., 1986), a more effective treatment for dental caries is needed.

*Bergenia crassifolia* (L.) Fritsch, belonging to the genus *Bergenia* (*Saxifragaceae*), is widely used in Poland, Ukraine, Russia, Mongalia, Siberia, and Xinjiang in China as a folk medicine or tea (Shikov et al., 2010, 2012). This plant is commonly known as badan, Siberian tea, Mongolian tea, leather bergenia, winter-blooming bergenia, heartleaf bergenia and elephant’s ears (Vereschagin et al., 1959). Extracts of roots and leaves from this plant have been reported to exert a variety of anti-inflammatory (Lee and Kim, 2012), anti-cancer (Popov et al., 2005), anti-diabetic, anti-viral (Churin et al., 2005), anti-diarrheal (Gammerman et al., 1984) and anti-septic (Kumar et al., 2012) bioactivities. The antimicrobial activity of the *B. crassifolia* leaf extract has been demonstrated against Gram-negative bacteria and Gram-positive cocci (Fedoseyeva et al., 2000). However, no study detailing effects of the *B. crassifolia* leaf on dental caries has been reported. The aim of this study was to explore a method that would be convenient and effective in preventing and curing dental caries.

## Materials and Methods

### Preparation of Extracts

Leaves of *B*. *crassifolia* (L.) Fritsch, collected from Xinjiang Uygur in the Autonomous Region, were dried at 25°C and ground in a grinder to a fine powder. The powdered material was extracted using three methods. First, 500 g of the powder was extracted by refluxing with 95% ethanol (w/v, 1:10). The extract solution was then filtered, and the solvent was evaporated on a rotary-evaporator under reduced pressure at 40°C to give the HP-95% ethanol extracts. Second, equal amounts of the powder were boiled with distilled water (w/v, 1:10), and the solutions were treated in the same way as the first to give the HP-water samples. Part of each of these samples was dissolved in 400 mL distilled water and transferred to a separator funnel and extracted with *n*-butyl alcohol to give the HP-BuOH sub-extracts. Third, an aliquot (500 g) of the powder was treated by refluxing through 70% ethanol and successively extracted with chloroform and ethyl acetate. The solvent extract was evaporated to dryness to give the HP-CHCl_3_ and HP-EtOAc sub-extracts. Finally, all samples were dissolved in dimethyl sulfoxide (DMSO).

### Bacterial Strain and Growth Conditions

*Streptococcus mutans* (ATCC 25175) provided by the Guangdong Microbiology Culture Center was cultured in brain heart infusion (BHI; Hopebio, Qingdao, China) broth containing 1% sucrose and incubated at 37°C for 24 h. After incubation, the concentration of bacteria was 10^7^ cfu/mL as determined by spectrophotometry (OD_630_ = 0.2). Strains were stored at -80°C in 20% glycerol for longer preservation and storage.

### Antimicrobial Activity Assay

The microdilution method was used to determine the potential inhibitory activity of extracts in this study as described previously (Palomino et al., 2002) with minor modifications. The extracts were two-fold diluted with BHI medium, with the final concentrations of extracts tested ranging from 2.5 to 0.156 mg/mL. The final concentration of DMSO was 1%, which was non toxic to bacteria (Figure 2 in Supplementary Data Sheet 1). Culture suspensions (100 μL per well) were added to 96-well polystyrene microplates, along with 100 μL of diluted extracts at different concentrations. Thereafter, twenty microliters of a sterile solution of resazurin sodium (1 mg/mL) was added per well. Non-treated with extracts bacteria and BHI broth medium were used as negative controls, and chlorhexidine, containing 1% metronidazole, at the final concentration of 0.6 mg/mL was used as the positive control. Finally, the 96-well polystyrene microplates were incubated at 37°C for 24 h. The lowest concentration of extracts which could prevent the solution from changing from blue to pink was the minimal inhibitory concentration (MIC).

The minimal bactericidal concentration (MBC) was determined with the BHI agar dilution method. The bacterial cultures from the wells with test samples at concentrations equal to or higher than the MIC were transferred onto BHI agar plates and incubated for 24 h. The MBC was defined as the lowest concentration which resulted in no visible bacterial colonies on the agar plates after 24 h of incubation.

### XTT Reduction Assay

The 2,3-bis (2-methoxy-4-nitro-5-sulfophenyl)-5-[(phenyla-mino) carbonyl]-2H- etrazolium hydroxide (XTT) assay was used to evaluate the viability of bacterial cells within the biofilms as described previously (Huang et al., 2012) with some modifications. The extract of *B. crassifolia* leaves was diluted in BHI broth containing 1% sucrose to final concentrations equal to the MIC, 1/2 MIC, 1/4 MIC and 1/8 MIC. 0.6 mg/mL chlorhexidine containing 1% metronidazole was used as the positive control, and the bacteria medium was used as the negative control. XTT reagent solution was mixed with XTT solution (0.5 mg/mL in PBS, Sigma, St. Louis, MO, United States) and menadione solution (1 μmol/mL) in acetone (Sigma). The ratio of volumes of the XTT solution and menadione solution was 50:1. One hundred microliters of bacteria culture and an equal quantity of BHI broth were added in flat-bottomed 96-well plates for 24 h at 37°C. After incubation, supernatants were removed, and the wells were gently washed three times with 200 μL of sterile PBS (pH 7.0) to remove planktonic and non-adherent cells. The cultures were then incubated at 37°C for another 22 h of growth in 200 μL of BHI broth including 100 μL of test extracts at various concentrations. Subsequently, planktonic cells were discarded, and weakly adherent cells were detached by gently rinsing twice with PBS (pH 7.0). One hundred microliters of XTT reagent was added, and then the plates were kept in the dark for 2 h at 37°C. After incubation, the absorbance of the supernatants was detected at 490 nm using an ELISA reader.

### Cell Damage Assay

LDH assay was conducted to determine the bacterial cell damage within the biofilm. Briefly, 100 μL bacterial culture together with 100 μL BHI broth were added into 96-well plates and incubated for 24 h at 37°C. After incubation, supernatants were discarded, and the wells were washed three times with sterile PBS. Thereafter, 200 μL of test samples at different concentrations (1/8 MIC to MIC) were added and incubated for a further 24 h at 37°C. Finally, the supernatants were collected and used for LDH activity detection. The bacteria culture and BHI broth were used as a negative control. LDH activity was measured using a Lacate Dehydrogenase Assay kit (Sigma), and the absorbance was detected at 450 nm.

### Extracellular Polysaccharide (EPS) Production Assay

The method for determining the inhibition of EPS production was carried out as described previously (Packiavathy et al., 2012) with some modifications. Freshly grown bacterial culture (500 μL) was added to 5 mL of sterile BHI broth with or without test samples at final concentrations ranging from 1/8 MIC to MIC. After incubation at 37°C for 16 h, cultures were centrifuged at 4°C for 30 min at 12,000 × *g*. After the supernatant was removed, the precipitate was resuspended with sterile water and then centrifuged again. The supernatant collected from both centrifugations contained the water-soluble glucans. The precipitate was resuspended with NaOH (0.1 mol/L) and centrifuged, and the supernatant that was collected contained the water-insoluble glucans. The supernatant was filtered through 0.22-μm nitrocellulose membrane filters. Three volumes of chilled 95% ethanol was added to the filtered supernatant and incubated overnight at 4°C to precipitate the EPSs. The quantification of EPSs was conducted using the phenol/H_2_SO_4_ method as described previously (Dubois et al., 1956). Briefly, for EPS quantification, one volume of ice-cold 5% phenol and five volumes of concentrated sulphuric acid were added to one volume of EPS solution to develop a red color. The absorbance of the color was detected at 492 nm. The percent inhibition of biofilm viability was calculated by using the following formula.

$$\begin{array}{c} \mathrm{Percentage}\;\mathrm{of}\;\mathrm{inhibition}=\;{\left[(\mathrm{control}\;{\mathrm{OD}}_{492}\;-\;\mathrm{treated}\;{\mathrm{OD}}_{492})/control\;{\mathrm{OD}}_{492}\right]}^{*}100\% \end{array}$$

### GTF Activity Assay

The method for producing the crude extract of Gtfs was used as described by Koo et al. (2000) with some modifications. *S. mutans* (20 mL) in the logarithmic phase of growth was incubated in 200 mL BHI broth containing 1% sucrose at 37°C for 24 h. After incubation, the culture was removed and centrifuged (4°C, 12000 × *g*, 30 min). The supernatant was collected and treated with ammonium sulfate at 60% saturation for 24 h. The precipitate was dialyzed for 48 h against PBS (pH 6.8) which contained 1 mM phenylmethylsulfonyl fluoride (PMSF) as a protease inhibitor. The dialyzed preparation was used as the crude extracellular Gtfs and stored at -20°C. The crude Gtfs were dissolved with PBS (pH 6.0), and the water-insoluble glucans formed by Gtfs were measured as described previously (Ooshima et al., 2000) with some modifications. The reaction mixtures contained 1 mL of sterile 0.1 M sucrose as a substrate and 200 μL of crude Gtfs. The *B. crassifolia* leaf extracts (200 μL) at different concentrations ranging from 1/4 MIC to MIC were assayed for the inhibition of synthesis of water-insoluble glucans. Following incubation at 37°C for 18 h, the quantity of water-insoluble EPS was determined using the method as described above.

### Live/Dead Bacteria Staining

An aliquot of *S. mutans* (100 μL) and an equal volume of BHI broth were added in each well of a 96-well microplate and incubated at 37°C for 24 h. After incubation, the supernatants of the wells were removed, and the bacteria were washed once in PBS (pH 7.4). Samples were added at the final concentration of 1/2 MIC for incubation at 37°C for 18 h. Cultures were gently removed, washed twice with sterile water and stained using a Live/Dead^®^BacLight^TM^ Bacterial Viability Kit (L13152, Invitrogen, Carlsbad, CA, United States) for 30 min at room temperature in the dark. Stained cells were observed under a fluorescence microscope (Nikon Eclipse 80i; Nikon Co., Japan). SYTO9, which is membrane-permeable and will stain all cells, can be detected by green fluorescence. PI, which is membrane-impermeable and stains cells with damaged membranes, gives red fluorescence. Twenty fields were analyzed for each treatment.

### Cytotoxicity Assay

Human oral epithelial cells (HOECs) were cultured in Dulbecco’s Modification of Eagle’s medium (DMEM) containing 10% FBS and incubated at 37°C in a humidified atmosphere of 5% CO_2_.

The 3-(4,5-dimethylthiazol-2-yl)-2,5-diphenyltetrazolium bromide (MTT, Sigma–Aldrich, St. Louis, United States) assay was performed as described previously (Mosmann et al., 1986). Briefly, cells were seeded into 96-well plates at a density of 1 × 10^5^ cells per well. After incubation at 37°C and 5% of CO_2_ for 24 h, cells were treated with various concentrations (0.005, 0.05, 0.5, and 5 mg/mL) of extracts/sub-extracts for 44 h. Afterwards, 20 μL of MTT solution were added into each well and incubated further for 4 h. After removing the medium, the formazan crystals were dissolved in DMSO, and the absorbance was measured at 570 nm on a micro-plate reader. Three independent experiments for both cell lines were conducted. The inhibition percentage of biofilm viability was calculated by using the following formula.

$$\begin{array}{c} \mathrm{Percentage}\;\mathrm{of}\;\mathrm{inhibition}\;=\;{\left[(\mathrm{control}\;{\mathrm{OD}}_{570}\;-\;\mathrm{treated}\;{\mathrm{OD}}_{570})/control\;{\mathrm{OD}}_{570}\right]}^{*}100\%. \end{array}$$

### Statistical Analysis

All experiments were performed in triplicate and repeated at least three times independently. One-way analysis of variance was performed to detect the significant effects of variables, followed by the Student-Newman-Keuls *post hoc* test and Student’s *t*-test. Differences were considered significant for *P-*values < 0.05. A line relationship was observed when *P-*values < 0.05 and *R*^2^> 0.9. Statistical analysis was performed using Excel software, version 16.0 (Chicago, IL, United States).

## Results

### Antimicrobial Activity of *B. crassifolia* Leaves

**FIGURE 1:**
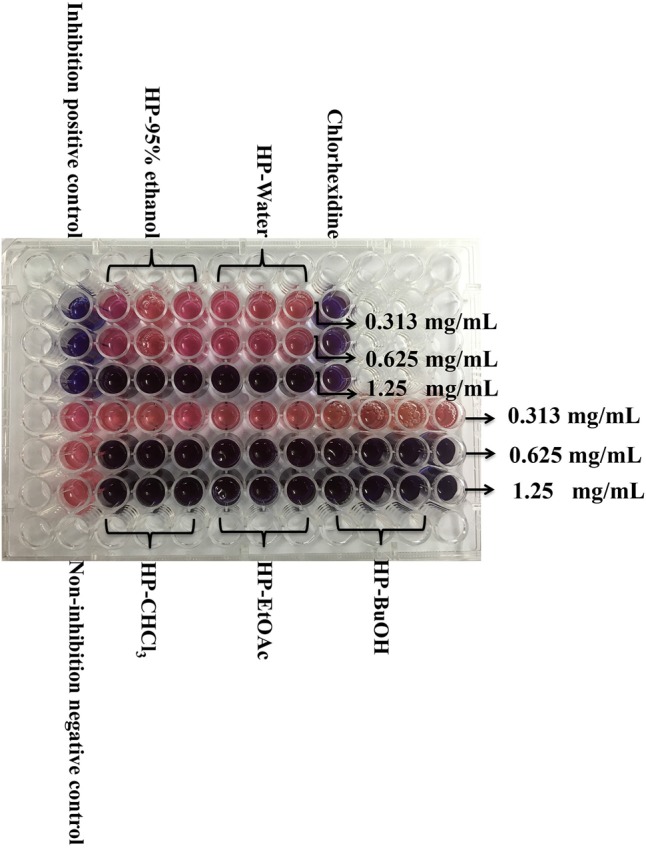
Determination of MIC for extracts/sub-extracts against *S. mutans.* After incubation with resazurin sodium 24 h, the lowest concentration that could protect the color from changing to pink was determined as the MIC. Other changing color pictures are displayed in Supplementary Data Sheet 2.

The resazurin microdilution method was used to determine the MIC, which could react with live bacteria and change the culture color from blue to pink. As shown in **Figure 1**, the MIC values of the HP-95% ethanol and HP-water samples were both 1.25 mg/mL, and those of HP-CHCl_3_, HP-EtOAc and HP-BuOH were all 0.625 mg/mL. The MBC values determined by the agar dilution method were shown to be 2 or 3 times higher than the MIC values (shown in **Table 1**), which is consistent with a previous report indicating that the MBC values were typically two to four times higher than the MIC values (Song et al., 2006). These results suggest that the extracts of *B. crassifolia* (L.) Fritsch leaves all exhibited inhibitory activities against *S. mutans*.

**Table 1:** Minimum inhibitory concentration (MIC) and the minimum bactericidal concentration (MBC) values of *B. crassifolia* leaf extracts/sub-extracts against *S. mutans* ATCC 25175.

	Species/strain	
	*S. mutans* (ATCC 25175)	
Extracts/sub–extracts	MIC (mg/mL)	MBC (mg/mL)
HP–95% ethanol	1.25	2.5
HP–Water	1.25	2.5
HP–CHCl_3_	0.625	1.25
HP–EtOAc	0.625	1.67
HP–BuOH	0.625	1.67

### Effects of *B. crassifolia* Leaves on Biofilms

**FIGURE 2:**
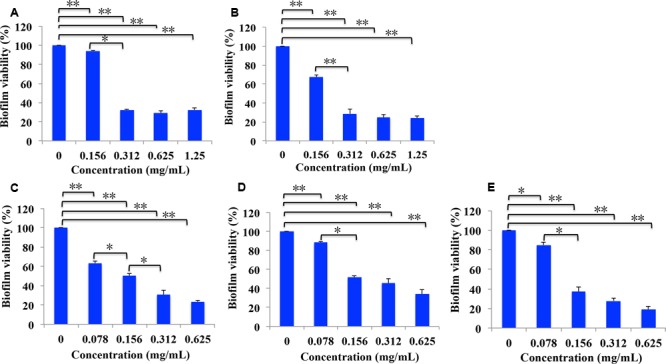
Percentage of bacterial viability within biofilms measured by the XTT assay. **(A)** Extracts of 95% ethanol, **(B)** extracts of water, **(C)** sub-extracts of EtOAc, **(D)** sub-extracts of CHCl_3_, **(E)** sub-extracts of BuOH. Data are presented as the mean + standard deviation based on three individual tests. *^∗^P* < 0.05 and *^∗∗^P* < 0.01.

As cariogenic biofilms play an important role in the development of caries, we then evaluated the effect of *B. crassifolia* leave extracts on biofilms. *S. mutans* was grown in 96-well polystyrene plates for 24 h. The non-adherent bacteria were then removed, and the extracts were added to treat the adherent bacteria for another 24 h, followed by the XTT reduction assay to detect the viability of *S. mutans* within biofilms. As shown in **Figure 2**, the viability of *S. mutans* within biofilms decreased significantly upon treatment with all extracts/sub-extracts but with different sensitivities. Compared with control groups, the percentages of remaining bacteria in the biofilms were initially reduced in proportion to the concentration of extracts and then entered a stable phase at a higher level. In addition, the maximum inhibition was not significantly different among the test samples (∼66–80%, *P* > 0.05). These results indicated that *B. crassifolia* leaves could effectively inhibit the viability of biofilms of *S. mutans.*

### Effects of *B. crassifolia* Leaves on Bacterial Cells within Biofilms

**FIGURE 3:**
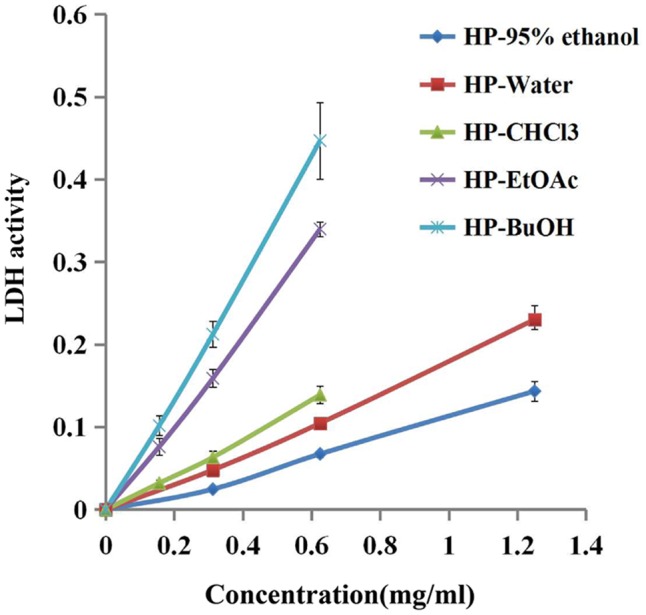
Bacterial cell damage within the biofilm based on LDH activity in different test samples. The extracts of *B. crassifolia* leaves were serially diluted twofold in BHI broth containing 1% sucrose from 1/8 MIC to MIC respectively. The graph represents the actual concentrations of the MIC dilutions.

LDH is an intrinsic intracellular enzyme of *S. mutans*, which catalyzes pyruvic acid to synthesize lactic acid. To test the possibility that *B. crassifolia* leaves could decrease the viability of bacteria within biofilms, we examined LDH activity in the supernatant, which is only detected in the extracellular matrix when the bacterial cell membrane is not intact. The results as shown in **Figure 3** indicated that LDH activities in supernatants are increased upon treatment of all extracts at concentrations from 1/8 MIC to 1/2 MIC (*P* < 0.05). A linear relationship was observed between LDH activities and the concentration of test samples, and the R^2^ values of extracts and sub-extracts were all greater than 0.99. When comparing among the test samples at the concentration of 1/2 MIC, the HP-BuOH sub-extract led to the highest LDH activity, while the HP-CHCl_3_ sub-extract led to the lowest. The results indicate that the extracts of *B. crassifolia* leaves could damage the bacterial cell membrane to kill the bacteria within biofilms, which is one mechanism of biofilm reduction by the test samples.

### Effects of *B. crassifolia* Leaves on EPS Production

**FIGURE 4:**
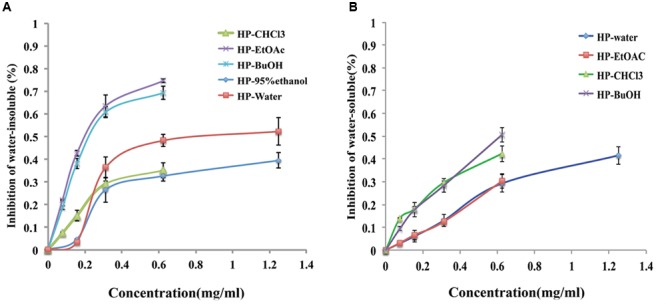
Percent inhibition of extracts/sub-extracts effect on EPS glucans by the phenol/H_2_SO_4_ method. **(A)** (%) Inhibition of water-insoluble glucans. **(B)** (%) Inhibition of water-soluble glucans. The extracts of *B. crassifolia* leaves were serially diluted twofold in BHI broth containing 1% sucrose from 1/8 MIC to MIC respectively. The graph represents the actual concentrations of the MIC dilutions. Data are presented as the mean ± standard deviation.

Extracellular polysaccharides are essential virulence factors for *S. mutans* to adhere to the surface of the tooth and form cariogenic biofilms, which are secreted by Gtfs. The phenol/H_2_SO_4_ method was used to determine the reduction of EPSs produced by *S. mutans* that were treated with extracts/sub-extracts of *B. crassifolia*, and the results are shown in **Figure 4**. Compared with the untreated control, the extracts/sub-extracts exhibited significant inhibitory effects on water-insoluble EPSs (*P* < 0.01, **Figure 4A**). The inhibition rates by the HP-95% ethanol and HP-water test samples increased as the concentrations increased from 1/8 MIC to 1/2 MIC, while the inhibition was not different between 1/2 MIC to MIC. Sub-extracts also showed significant inhibitory activities against water-insoluble EPSs, which rose as the concentration increased (*P* < 0.01). This data, together with those shown in **Figures 2, 3**, suggest that the extracts of *B. crassifolia* leaves could decrease adhesion and viability of *S. mutans* and thus prevent biofilm formation or maturation.

Water-soluble EPSs are energy supplements for bacteria within biofilms. The same method as above was used to measure the quantity of water-soluble EPSs, and the results are presented in **Figure 4B**. The inhibition rates by the HP-water, HP-CHCl_3_, HP-EtOAc and HP-BuOH extracts/sub-extracts were significantly increased as the concentration was increased from 1/8 MIC to MIC, while the inhibition of extracts produced by 95% ethanol was 11% at only the MIC (1.25 mg/mL). A linear relationship was observed between the inhibitory activity and concentrations of test samples, and the *R*^2^ values of the HP-water, HP-CHCl_3_, HP-EtOAc and HP-BuOH samples were 0.95, 0.93, 0.99, and 0.98, respectively. Therefore, these results demonstrated that most extracts of *B. crassifolia* leaves could effectively decrease the quantity of water-soluble EPSs, which would restrict the source of metabolizable carbohydrates for biofilm formation.

### Effects of *B. crassifolia* Leaves on Gtf Activity

**FIGURE 5:**
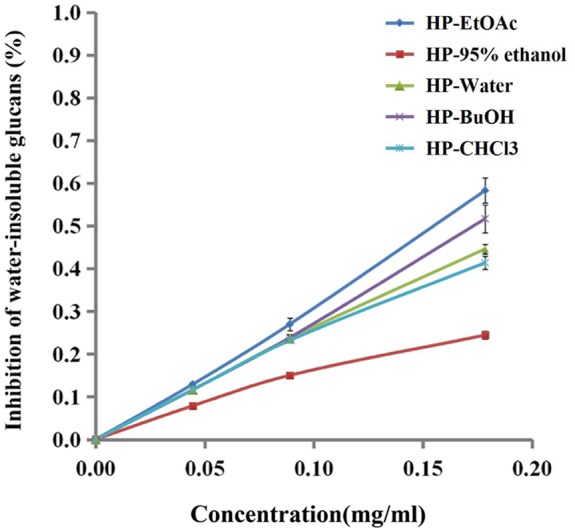
Effect of *B. crassifolia* leaf extracts on water-insoluble glucan formation by Gtfs of *S. mutans* from sucrose. The formation of water-insoluble glucans was expressed as a percent of the control (without sample).

Gtfs, which are key enzymes for forming biofilms, can synthesize water-insoluble glucans with sucrose and attach the bacteria onto the surface of the tooth. As shown in **Figure 5**, the extracts/sub-extracts exerted inhibitory activity on Gtfs, resulting in a significant decrease in the synthesis of water-insoluble glucans (*P* < 0.01). The inhibition of test samples was dependent on their concentrations, showing a linear relationship between the inhibition rate and concentration of extracts/sub-extracts. The *R*^2^ values for the HP-EtOAc, HP-95% ethanol, HP-water, HP-BuOH and HP-CHCl_3_ samples were between 0.98 and 0.99. Combined with the data in **Figure 4**, these results suggest that the extracts of *B. crassifolia* leaves could prevent adhesion or aggregation of the bacteria to form a biofilm through suppressing Gtfs to synthesize EPSs.

### Effects of *B. crassifolia* Leaves on Biofilm Structure

**FIGURE 6:**
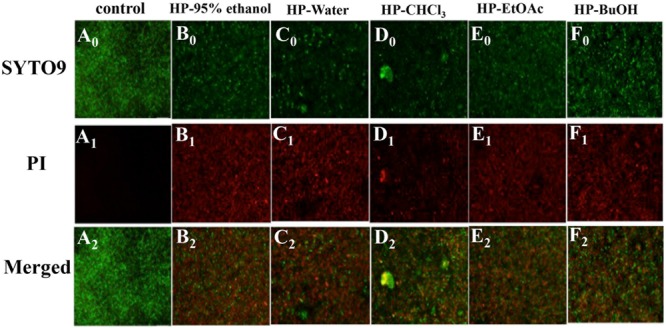
Effects of *B. crassifolia* leaf extracts on *S. mutans* ATCC25175 biofilm by using a fluorescence microscope. Live cells exhibited green fluorescence (SYTO 9), whereas bacteria with damaged membranes exhibited red fluorescence (PI). **(A)** control; **(B)** HP-95% ethanol; **(C)** HP-Water; **(D)** HP-CHCl_3_; **(E)** HP-EtOAc; **(F)** HP-BuOH, and **(0)** stained with SYTO9; **(1)** stained with PI; **(2)** Merged.

The Live/Dead^®^BacLight^TM^ Bacterial Viability Kit was used to visualize the viability of microorganisms within the biofilms. The kit included two types of stains: SYTO 9, which stains live bacterial cell with green fluorescence, and PI, which stains cells with impaired membrane activity with red fluorescence. Meanwhile, a yellow color indicated overlapping images of live and dead bacteria. The test samples were effective on the biofilms as shown in **Figure 6**. Compared with the control group, the biofilm treated with extracts/sub-extracts were thin and unable to form bacterial colonies, showing large amounts of red stained dead cells. This result further confirms the inhibitory effects of the extracts of *B. crassifolia* leaves on formation or maturation of biofilm.

### Cytotoxicity of *B. crassifolia* Leaves to Oral Cells

Finally, we also evaluated the cytotoxic effects of all extracts and sub-extracts on HOECs. The IC_10_ value commonly represents a non-cytotoxic concentration. As shown in **Table 2**, the IC_10_ values of extracts/sub-extracts in HOECs were higher than their MIC, while HP-water extracts showed no cytotoxicity up to the concentration of 5 mg/mL. This data suggest that the extracts of *B. crassifolia* leaves could inhibit biofilm formation of oral pathogenic bacteria without cytotoxicity to oral cells.

**Table 2:** IC_10_ values of *B. crassifolia* leaf extracts/sub-extracts in HOECs.

	HOECs
Extracts/sub-extracts	IC_10_ (mg/mL)
HP–95% ethanol	1.31
HP–Water	>5.00
HP–CHCl_3_	1.42
HP–EtOAc	1.18
HP–BuOH	0.94

## Discussion

Dental plaque is an oral bacterial biofilm and the critical factor causing dental caries. As biofilms increase the bacterial resistance to drugs, inhibiting or damaging them is key to preventing and curing dental caries. *S. mutans* is one of the primary microorganisms that adhere on the tooth surface. Many studies have focused on the individual role of *S. mutans* in single-species biofilms on dental caries (Xu et al., 2012; Zhang et al., 2013). As above, *S. mutans* biofilms have been widely used as a model of dental caries for studying the biofilm mechanism or drug screening (Hwang et al., 2014). The findings of the present study indicated that the test samples influence the viability of *S. mutans* within biofilms. The results of the XTT reduction assay and live/dead bacteria staining assay showed that the biofilm are decreased when treated with the leaf extracts (**Figures 2, 6** and Figure 1 in Data Sheet 1). In addition, the extracts could also damage the integrity of bacterial cells within biofilms, which led to the leakage of LDH that is an intrinsic intracellular enzyme (**Figure 3**). Chlorhexidine, as a broad-spectrum antibiotic, is widely used to prevent or cure oral diseases. As a positive control in this study, chlorhexidine decreased the biofilms to 43%, while the reduction by the HP-water, HP-EtOAc and HP-BuOH samples from B. crassifolia (L.) Fritsch leaves at the same concentrations were more significant (*P* < 0.05) at approximately between 65% and 80%.

Biofilm formation is a dynamic process that basically includes three successive steps, adhesion, aggregation and maturity. Adhesion is a prerequisite to forming the biofilm and contributes to both biofilm development and maturation (Marsh, 2006; Liu et al., 2016). In this study, we explored the influence of plant extracts on Gtf enzymes of *S. mutans* to illuminate the mechanism of inhibiting biofilm formation. Gtf enzymes, among the most important virulence factors in biofilm formation, are secreted by *S. mutans* to synthesis EPSs with sucrose. EPSs essentially contribute to the formation, adherence and structural integrity of the dental biofilm (Paes Leme et al., 2006). They accumulate together with bacteria cells on the tooth surface and subsequently form micro-colonies through an EPS-mediated process, leading to biofilm formation (Koo et al., 2010). Furthermore, the presence of an EPS-rich matrix and microcolonies is critical for the biofilm physical integrity and attachment strength at the pellicle interface (Vinogradov et al., 2004; Xiao et al., 2012; Hwang et al., 2014; Waters et al., 2014; Kim et al., 2015). The results of the current study suggest that the inhibitory effects of the extracts could reduce biofilm adhesion through inhibiting Gtfs from synthesizing EPSs (**Figures 4, 5**). Moreover, inhibitory effects of extracts/sub-extracts from *B. crassifolia* leaves on the water-insoluble glucans were more potent than on water-soluble glucans. Compared with chlorhexidine, the inhibition by the HP-water extract on water-insoluble glucans was similar at approximately 50%.

Based on results above, our study demonstrated that the test samples can be antimicrobial and inhibit biofilm formation. Actually, extracts from *B. crassifolia* leaves have been reported to elicit antimicrobial activity against *Pseudomonas aeruginosa* with MIC of 15.63mg/mL, *Bacillus cereus, Escherichia coli*, and *Staphylococcus aureus* with MIC of 62.50 mg/mL (Kokoska et al., 2002). Compared with MIC against *S. mutans* (**Table 1**), obviously, extracts of *B. crassifolia* leaves showed more potent inhibitory effect on *S. mutans*. However, low toxicity is always desirable for antimicrobial agents to be utilized in the mouth. Chlorhexidine, as a general antibiotic and mouthwash, has been reported to have toxic effects on epithelial cells and even more so on the viability of stem cells. In contrast, we confirmed that the extracts of *B. crassifolia* (L.) Fritsch leaves showed no toxicity against oral epithelial cells at the same concentration as the MIC, and the HP-water extracts seemed to have the safest profile (**Table 2**). Therefore, *B. crassifolia* (L.) Fritsch leaves have the potential to be developed as a drug or mouthwash to prevent or cure dental caries. In conclusion, the results of the present study support that the leaves of *B. crassifolia* (L.) Fritsch could prevent and cure dental caries through two mechanisms. One pathway was by directly inhibiting the viability of planktonic and sessile bacteria, and the other was by inhibiting the ability of Gtf enzymes to synthesize EPSs, thereby decreasing adhesion and biofilm formation. The above *in vitro* experiments confirmed that administration of extracts of the *B. crassifolia* plant would be more convenient to than as a traditional medicine. However, this study only used *S. mutans* as the single-species biofilm model, and we will need to further confirm the antibiofilm activity of extracts/sub-extracts from *B. crassifolia* leaves on cariogenic polymicrobial biofilms, both *in vitro* and *in vivo*.

## Author Contributions

YcL, contributed to conception, design, data acquisition, analysis, and interpretation, drafted and critically revised the manuscript; YW, LS, HH, and YxL, contributed to conception, design, data acquisition, analysis, and interpretation, drafted and critically revised the manuscript; JY, YB, CY, YH, YX, QS, FW, LL, XY, and HM, contributed to conception, data analysis and interpretation, critically revised the manuscript.

## Conflict of Interest Statement

The authors declare that the research was conducted in the absence of any commercial or financial relationships that could be construed as a potential conflict of interest.
