# Supplementary material for: Anti-biofilm Activities from Bergenia crassifolia Leaves against Streptococcus mutans
Source: Front Microbiol. 2017 Sep 13;8:1738. doi: 10.3389/fmicb.2017.01738 (PMC5601420; doi:10.3389/fmicb.2017.01738)
Supplement: Supplementary file 1 [file Data_Sheet_1.ZIP › Supplement figures/Figure legends.docx]

Figure 1. Percent inhibition of extracts/sub-extracts effect on bacterial adherence to a glass surface as detailed in Duarte et al. Briefly, After incubation, the adhering cells were washed and resuspended in an ultrasonic bath. The amount of adherent cells was measured spectrophotometrically at 550 nm.

Figure 2．Effect of DMSO and extracts/sub-extracts of *Bergenia crassifolia* leaves on the growth of *S. mutans*, and the concentration of test samples were 1/4 MIC.
