# Supplementary figures and images for: Anti-biofilm Activities from Bergenia crassifolia Leaves against Streptococcus mutans
Source: Front Microbiol. 2017 Sep 13;8:1738. doi: 10.3389/fmicb.2017.01738 (PMC5601420; doi:10.3389/fmicb.2017.01738)

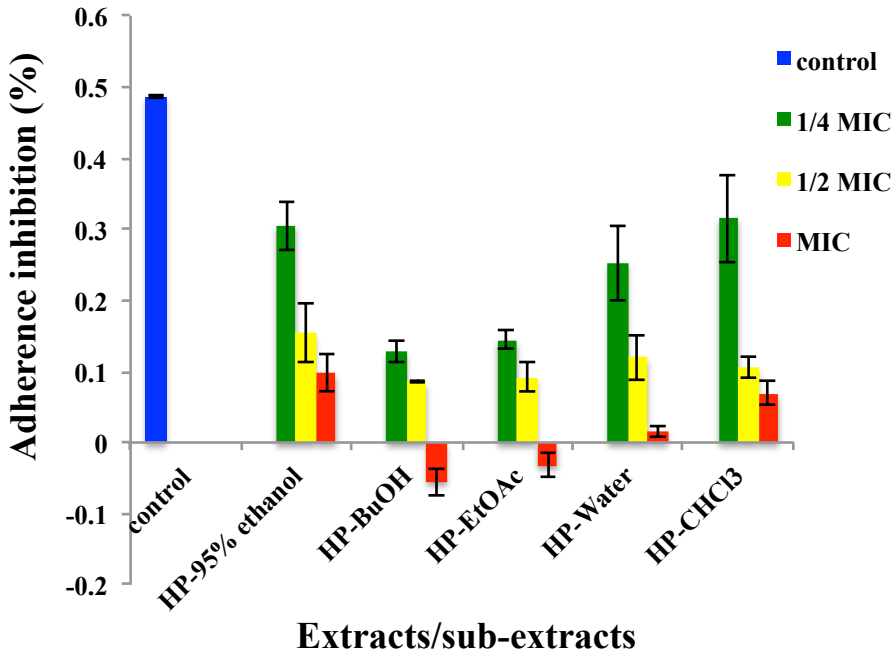

Supplement: Supplementary file 1 [file Data_Sheet_1.ZIP › Supplement figures/figure 1.pdf]

**OD Values**

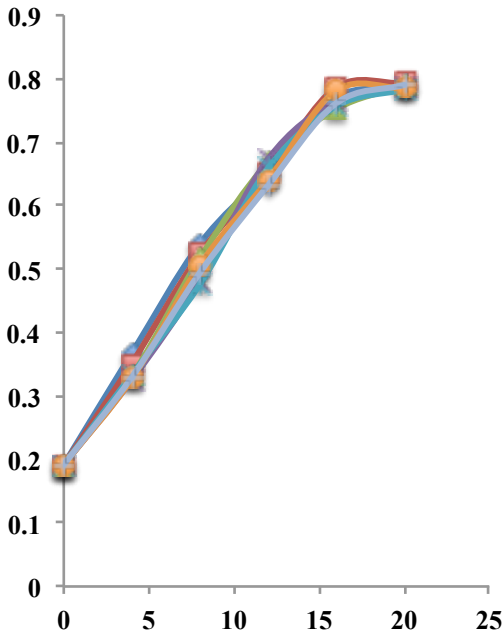

**Time (h)**

- bacteria
- DMSO
- HP-95% ethanol
- HP-Water
- HP-CHCl<sub>3</sub>
- HP-EtOAc
- HP-BuOH

Supplement: Supplementary file 1 [file Data_Sheet_1.ZIP › Supplement figures/figure 2.pdf]

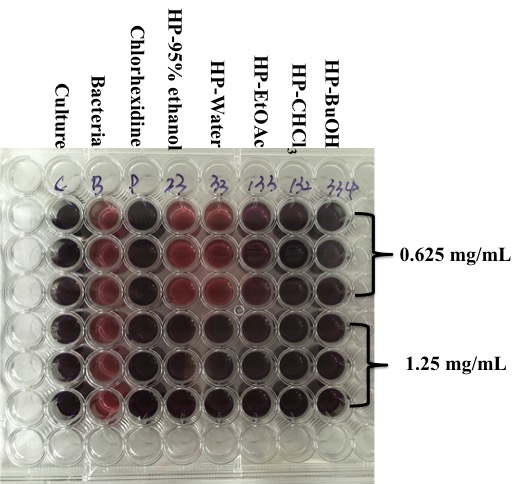


The same color represents that one concentration was performed in triplicate.

Supplement: Supplementary file 2 [file Data_Sheet_2.DOCX]
